# Use of multikinase inhibitors/lenvatinib concomitant with antiresorptive therapy for bone metastases from radioiodine‐resistant differentiated thyroid cancer

**DOI:** 10.1002/cam4.4983

**Published:** 2022-07-03

**Authors:** Elena Navarro‐Gonzalez

**Affiliations:** ^1^ Department of Endocrinology and Nutrition University Hospital Virgen del Rocío Sevilla Spain

**Keywords:** bisphosphonates, bone metastases, denosumab, lenvatinib, multikinase inhibitors, radioiodine‐refractory thyroid cancer

## Abstract

Bone is the second most common distant metastasis site in differentiated thyroid cancer (DTC) and is normally associated with the presence of other metastases, which are usually radioiodine‐resistant. The presence of bone metastasis (BM) determines low survival and greater morbidity due to the frequency of skeletal‐related events (SREs), which can be a serious complication of BM. There is evidence that antiresorptive therapy (AT) reduces SREs in other solid tumors, but not yet in DTC BM, for which data are scant. The same is true for systemic therapy with multikinase inhibitors (MKIs). In general, the results for MKI use are well known, although the effect on BM has rarely been evaluated. While MKIs are indicated in current clinical practice guidelines, studies evaluating the benefits and risks of concomitant treatment with MKIs and AT are lacking, and the available data come from small samples in retrospective studies. The objective of this article is to review the latest evidence for concomitant MKIs and AT.

## Abstract 2

Bone is a common distant metastasis in differentiated thyroid cancer, that is associated with the presence of other metastases, low survival and high morbidity. There is evidence that antiresorptive therapy and multikinase inhibitors reduce skeletal‐related events in other solid tumors, but studies evaluating the benefits and risks of concomitant treatment are lacking in differentiated thyroid cancer. This article reviews the latest evidence.

## 1 INTRODUCTION

In differentiated thyroid cancer (DTC), bone metastasis (BM) occurs in 2%–13% of cases,^1^ and is normally associated with the presence of metastases in other locations. Two‐thirds of metastatic patients do not achieve a complete response with radioiodine^2^ and, consequently, the absence of uptake of radioiodine (radioiodine‐resistant [RR]‐DTC) in BM is observed in up to 50% of patients,^3^ meaning that most will require specific anti‐tumor treatment with multikinase inhibitors (MKIs) to control disease progression.^4^, ^5^, ^6^

The presence of BM results in major morbidity, quantified by skeletal‐related events (SREs), which include pathological fractures, medullary compression, the need for external radiotherapy or surgery to control pain, spinal cord compression, and malignant hypercalcemia. These SREs, occurring in between 55% and 78% of patients with DTC and BM,^3^, ^7^ are associated with a greater risk of disease‐specific mortality and constitute a poor prognostic indicator compared to other distant metastases.^7^ Treatment with antiresorptive therapy (AT) can reduce or delay the onset of SREs and, therefore, in the group of patients with RR‐DTC and BM, concomitant therapy with MKIs and AT may be considered.

Provided both approaches have demonstrated improved morbidity and mortality of RR‐DTC patients as individual therapies, it is expected that their combination will result in even better overall disease control. However, to date, no evidence has been published on the use of this combination that could unequivocally support its use in RR‐DTC patients. Consequently, this paper reviews the currently available information on the use of each strategy for the management of BM from RR‐DTC and the potential benefits associated with their combined use.

## 2 CURRENT SITUATION

Multiple MKIs have been studied in recent decades, although the only ones currently approved for the treatment of RR‐DTC are lenvatinib, sorafenib, and cabozantinib. The SELECT study of lenvatinib shows a median progression‐free survival (PFS) of 14.7 months longer than for placebo,^8^ and the DECISION study of sorafenib, with a similar design, demonstrated an improvement of 5 months in median PFS over placebo.^9^ With regard to the specific anti‐tumor effect on BM, only limited information for lenvatinib is available. In the SELECT study, the effects of lenvatinib on BM showed a 51% objective response rate (ORR) in bone evaluated by an independent radiological committee. This indicates that lenvatinib is effective in controlling tumors in this location.^10^

AT blocks the increase in reabsorption and reduction in bone formation associated with BM whereby, if osteolysis is interrupted, the lesions can stabilize and the onset of SREs can be prevented and/or delayed. Two classes of antiresorptive drugs are currently approved for the treatment of BM, bisphosphonates (zoledronic acid, pamidronate) and denosumab. Bisphosphonates induce osteoclast apoptosis and reduce bone reabsorption, while denosumab is a monoclonal antibody that binds to the nuclear factor kappa‐B [RANK] ligand, and impairs its interaction with the RANK receptor, which inhibits osteoclast activity. Both drug classes have been approved for the treatment of BM from different types of tumors (notably breast, prostate, and multiple myeloma), since they reduce the incidence and delay the time to onset of the first SRE. These results have been extensively documented in randomized, controlled clinical trials.^11^ However, there is hardly any evidence of the effect of AT in DTC BM, since few studies have evaluated this specifically. The first study for which there are data is a trial of pamidronate in a series of 10 patients, demonstrating a reduction in bone pain from skeletal metastases, improvement in quality of life, and a partial radiologic response in 2 of 10 patients.^12^ The second retrospective study comprises a series of 50 patients and shows that zoledronic acid reduced the incidence of SREs as well as delayed the time to onset of the first SRE (50% in non‐treated vs. 14% in treated patients).^13^ Thus, zoledronic acid can also be given although its greater potency may increase the risk of hypocalcemia and osteonecrosis of the jaw (ONJ), although this remains a rare complication. The same research group evaluated the effect of zoledronate in a prospective study of 19 patients, comparing it with 16 historic control patients, and observed a lower incidence of medullary compression.^14^

Denosumab is also used for the prevention of SREs in patients with bone metastases from solid tumors. Thus, denosumab is an alternative to bisphosphonate therapy in the management of thyroid cancer patients with BM. Although studies conducted in BM of solid tumors have shown the superiority of denosumab over zoledronate with regard to delaying or preventing SREs, no significant differences have been found in terms of overall survival or PFS.^15^, ^16^ These studies do not include patients with DTC, so there is no evidence of benefit of one drug over another specifically in DTC. Regarding the timing of AT, while there is no evidence to suggest that all patients with BM should start AT as soon as BM has been diagnosed and there is no tool to predict who will develop SRE, the European Society for Medical Oncology (ESMO) 2020^17^ guidelines recommend that therapy be started at the time of BM diagnosis, regardless of the existence or severity of symptoms. The same recommendation can be assumed for BM of the DTC Although the ESMO guidelines recommend continuing treatment with AT indefinitely, there is a relationship between long‐term use of bisphosphonates and the appearance of atypical fractures of the femur, the risk being higher when AT use exceeds 5 years.^18^ Therefore it is not recommended to continue AT beyond 3 years, although the decision should be tailored to the individual, considering the patient's fracture risk, comorbidity, life expectancy, and therapeutic compliance. Regarding the recommended dosing, bisphosphonates are administered every 4 weeks; although the results of some trials suggest a similar efficacy for administration every 12 weeks, long‐term results are not yet available, so the ESMO guidelines recommend initial treatment every 4 weeks for 3–6 months for bisphosphonates and every 4 weeks for denosumab.^17^ A recent study has demonstrated that, in patients with DTC BM treated with radioiodine, survival was greater when combined with denosumab.^15^ However, bisphosphonates or denosumab are seldom used in clinical practice. In fact, a retrospective study assessing the real‐life management of BM in 143 patients with DTC demonstrated that only 22.4% were treated with AT (usually zoledronate).^16^ The limited number of studies in treated patients prevents full evaluation of the potential effect of AT in the prevention of SREs in BM from DTC, but this does not indicate that there are no benefits in these patients. For this reason, the ESMO guideline on bone health in cancer recommends treatment with AT for BM of all solid tumors, and the current DTC guidelines include treatment with AT for patients with multiple progressive and/or symptomatic BM to prevent SREs, alone or concomitantly with other systemic therapies.

Additional benefit should be expected for combination therapy with MKIs and AT as most BM patients who have shown benefits have received concomitant systemic therapy, although the possible benefits for MKIs and AT in combination are unknown. In published studies in renal carcinoma, concomitant treatment improved median survival by 16 months in a few cases,^17^ and in lung cancer, the combination of MKIs and AT improved median PFS and overall survival.^18^ There is minimal information about the combination of both therapies in RR‐DTC. A patient with RR‐DTC treated with four lines of MKIs (sorafenib, sunitinib, pazopanib, and lenvatinib) and two lines of AT (zoledronate and denosumab) for 3 years, in whom bone lesion stability was achieved, was described in 2017.^19^ A trial investigating the efficacy of lenvatinib combined with denosumab in the treatment of patients with BM (the LENVOS [NCT03732495] trial), whose objectives are to ascertain the rate of SREs, PFS, ORR, analgesic use, and quality of life, is currently in the recruitment phase.^20^

The potential risks of concomitant therapy with MKIs and AT include ONJ, which occurs in 1.3%–1.8% of patients treated with AT, with no significant difference in risk between bisphosphonates and denosumab. ^21^, ^22^ ONJ has been described in DTC in 2 of 22 patients treated with bisphosphonates.^13^ ONJ has also been described in a few patients treated with MKIs (sunitinib, sorafenib) when not combined with AT, ^23^, ^24^ with only one case of DTC in which treatment with lenvatinib, without a prior background of AT, led to the development of ONJ.^25^ Therefore, concomitant administration of AT and MKIs may increase the risk of developing ONJ. This has been verified in small and larger series comprising 120 patients (one with DTC), in which the prevalence of ONJ was 11% in patients taking concomitant therapy (most were taking sunitinib), with a risk 5–10 times greater and of earlier onset than in the control group (533 patients) who received AT alone.^26^ In 2019, a retrospective study of 15 patients with RR‐DTC treated with denosumab and tyrosine kinase inhibitors was published, with three of 15 patients developing ONJ.^27^ The risk factors for developing ONJ include tooth extractions during treatment and previous periodontitis or tooth infections.^21^, ^24^

## 3 TREATMENT RECOMMENDATIONS

Despite the absolute lack of published evidence to support this combination strategy in patients with RR‐DTC, extrapolated data from other tumor types have enabled the American Thyroid Association, European Thyroid Association, and National Comprehensive Cancer Network guidelines^4^, ^5^, ^6^ to recommend initiating treatment with MKIs (lenvatinib, sorafenib) in rapidly progressing or symptomatic RR‐DTC and to combine them with AT (bisphosphonates or denosumab) if there are BM to reduce or delay SREs, morbidity and mortality, and prolong PFS.

If concomitant therapy is initiated, caution must be exercised and prophylactic measures provided for possible adverse effects of these drugs, which may have synergistic effects. Evaluation of the oral cavity should be performed by a maxillofacial surgeon to assess the risk of ONJ. Dental examination should be performed and all dental treatments should be completed, particularly dentoalveolar surgery, before the therapy begins, as well as professional teeth cleaning and caries control. Effective oral hygiene is recommended and risk behaviors for oral health should be limited (such as smoking, using drugs, and drinking alcohol). If zoledronate is given, kidney function must be monitored as treatment with bisphosphonates may lead to kidney failure, although this is infrequent and reversible, and the dose should be titrated depending on renal function alterations.^28^ Altered glomerular filtration has been described in some patients with RR‐DTC.^29^ In addition, there is a greater risk of developing hypocalcemia if denosumab is given,^30^ so this must be monitored, and treatment amended as necessary. AT, antiangiogenic agents, or both should be stopped before dentoalveolar surgery, and periodic oral health checks should be performed during therapy.^21^, ^31^

## 4 CONCLUSION

The presence of BM in advanced RR‐DTC has a poor prognosis, with low survival and high morbidity. Although MKIs have demonstrated clinical results in RR‐DTC, their effects on BM control have barely been evaluated and would appear to be lower than those seen in metastases at other locations. Early use of AT may reduce and/or delay the onset of SREs and associated morbidity, although the available data in RR‐DTC are scarce, and their use is accepted in parallel with the outcomes obtained in other solid tumors.

There are no data about the potential benefits of combined therapy or the risks of this treatment, although there is discrete evidence regarding the greater frequency of ONJ when certain drug classes are combined. There is an ongoing clinical trial to evaluate the outcomes from lenvatinib plus denosumab in patients with RR‐DTC BM, which will undoubtedly provide important information in the future. Until then, despite seeming feasible and currently recommended by guidelines, special caution should be exerted when combining these approaches in clinical practice.

## FUNDING INFORMATION

The author received an honorarium payment from Eisai Farmacéutica SA in line with ICMJE guidelines.

## CONFLICT OF INTEREST

Dr Elena Navarro‐Gonzalez has received research funding, honoraria, and non‐financial or other support from Eisai Farmacéutica SA.

## Data Availability

Data sharing is not applicable to this article as no new data were created or analyzed in this study.

## References

[1] Muresan MM , Olivier P , Leclere J , Sirveaux F , Brunaud KM , et al. Bone metastases from differentiated thyroid carcinoma. Endocr Relat Cancer. 2008;15:37‐49. 18310274 10.1677/ERC-07-0229

[2] Durante C , Haddy N , Baudin E , et al. Long‐term outcome of 444 patients with distant metastases from papillary and follicular thyroid carcinoma: benefits and limits of radioiodine therapy. J Clin Endocrinol Metab. 2006;91(8):2892‐2899. 16684830 10.1210/jc.2005-2838

[3] Farooki A , Leung V , Tala H , Tuttle M . Skeletal‐related events due to bone metastases from differentiated thyroid cancer. J Clin Endocrinol Metab. 2012;97(7):2433‐2439. 22564664 10.1210/jc.2012-1169

[4] Haugen BR , Alexander EK , Bible KC , et al. American Thyroid Association Management guidelines for adult patients with thyroid nodules and differentiated thyroid cancer. The American Thyroid Association Guidelines Task Force on Thyroid Nodules and Differentiated Thyroid Cancer. 2016;26(1):1‐133. 10.1089/thy.2015.0020 PMC4739132 26462967

[5] Fugazzola L , Elisei R , Fuhrer D , et al. European thyroid association guidelines for the treatment and follow‐up of advanced radioiodine‐refractory thyroid cancer. Eur Thyroid J. 2019;8:227‐245. 31768334 10.1159/000502229 PMC6873012

[6] NCCN guidelines https://www.nccn.org/professionals/physician_gls/pdf/thyroid.pdf Version 3.2020_February 2 2021

[7] Choksi P , Papaleontiou M , Guo C , Worden F , Banerjee M , Haymart M . Skeletal complications and mortality in thyroid cancer: a population‐based study. J Clin Endocrinol Metab. 2017;102(4):1254‐1260. 28324052 10.1210/jc.2016-3906 PMC5460727

[8] Schlumberger M , Tahara M , With LJ , Robinson B , Brose MS , Elisei R et al. Lenvatinib versus placebo in radioiodine‐refractory thyroid cancer New Engl J Med 2015;372(7):621–630, Lenvatinib versus placebo in radioiodine‐refractory thyroid cancer 25671254 10.1056/NEJMoa1406470

[9] Brose MS , Nutting CM , Jarzab B , et al. Sorafenib in radioactive‐iodine‐refractory locally advanced or metastatic differentiated thyroid cancer: a randomized double‐blind phase 3 trial. Lancet. 2014;384:319‐328. 24768112 10.1016/S0140-6736(14)60421-9 PMC4366116

[10] Habra MA , Song JH , Rietschel P . Outcomes by site of metastases for patients with radioiodine‐refractory differentiated thyroid cancer treated with Lenvatinib versus placebo: results from a phase 3, randomized trial. Abstract 55 presented at 2015 international thyroid congress. Thyroid 2015; S1

[11] D'Oronzo S , Coleman R , Brown J , Silvestris F . Metastatis bone disease: pathogenesis and therapeutic options. Up to date on bone metastasis management. J bone. Oncologia. 2019;15100205:100205. 10.1016/j.jbo.2018.10.004 PMC6429006 30937279

[12] Vitale G , Fonderico F , Martignetti A , et al. Pamidronate improves the quality of life and induces clinical remission of bone metastases in patients of thyroid cancer. Br J Cancer. 2001;12:1586‐1590. 10.1054/bjoc.2001.1832 PMC2363684 11401309

[13] Orita Y , Sugitami I , Toda K , Manabe J , Fujimoto Y . Zoledronic acid in the treatment of bone metastases from differentiated thyroid carcinoma. Thyroid. 2011;1:31‐35. 10.1089/thy.2010.0169 21058881

[14] Orita Y , Sugitami I , Takao S , Toda K , Manabe J , Miyata S . Prospective evaluation of zoledronic acid in the treatment of bone metastases from differentiated thyroid carcinoma. Ann Surg Oncol. 2015;12:4008‐4013. 10.1245/s10434-015-4497-0 25762482

[15] Menshawy A , Mattar O , Abdulkarim A , Kasem F . Nasreldin3 N, Menshawy E et al denosumab versus bisphosphonates in patients with advanced cancers‐related bone metastasis: systematic review and meta‐analysis of randomized controlled trials. Support Care Cancer. 2018;26:1029‐1038. 29387997 10.1007/s00520-018-4060-1

[16] Farii A , Frazer A , Farahdel L , AlFayyadh F , Turcotte R . Bisphosphonates versus denosumab for prevention of pathological fracture in advanced cancers With bone metastasis: a meta‐analysis of randomized controlled trials. J Am Acad Orthop Surg Glob Res Rev. 2020;4(8):e20.00045. 10.5435/JAAOSGlobal-D-20-00045 PMC7418898 32769706

[17] Coleman R , Hadji R , Body JJ , Santini D , Chow E , Terpos E . Bone health in cancer: ESMO clinical practice guidelines. Ann Oncol. 2020;31:1651‐1663. 10.1016/j.annonc.2020.07.019 32801018

[18] Ma S , Goh EL , Jin A , et al. Long‐term effects of bisphosphonate therapy: perforations, microcracks and mechanical properties. Sci Rep. 2017;7:43399. doi:10.1038/srep43399 28262693 PMC5338252

[19] Wu D , Gomes Lima CJ , Moreau SL , et al. Improved survival after multimodal approach with 131I treatment in patients with bone metastasis secondary to differentiated thyroid cancer. Thyroid. 2019;29:971‐978. 31017051 10.1089/thy.2018.0582

[20] Mazziotti G , Formenti AM , Panarotto MB , et al. Real‐life management and outcome of thyroid carcinoma‐related bone metastases: results from a nationwide multicenter experience. Endocrine. 2018;59:90‐101. 29110129 10.1007/s12020-017-1455-6

[21] Wong E , Kapoor A . Does bone‐targeted therapy benefit patients with metastatic renal cell carcinoma? Translational Oncology. 2020;13(2):241‐244. 31869748 10.1016/j.tranon.2019.10.009 PMC6931200

[22] Cui X , Li S , Gu J , et al. Retrospective study on the efficacy of bisphosphonates in tyrosine kinase inhibitor‐treated patients with non‐small cell lung cancer exhibiting bone metastasis. Oncol Letter. 2019;18(5):5437‐5447. 10.3892/ol.2019.10870 PMC6781563 31612052

[23] Morelli S , Puxeddu E . Long term efficacy and safety of fourth‐line multikinase inhibitor treatment with lenvatinib in a young papillary thyroid carcinoma patient. Drugs Context. 2017;6:212310. 29167691 10.7573/dic.212310 PMC5699105

[24] Study of the Efficacy of Lenvatinib Combined With Denosumab in the Treatment of Patients With Predominant Bone Metastatic Radioiodine Refractory Differentiated Thyroid Carcinomas (LENVOS). Available at: https://clinicaltrials.gov/ct2/show/results/NCT03732495

[25] Ruggiero SL , Dodson TB , Fantasía J . American association of oral and maxilofacial surgeons position paper on medication‐related osteonecrosis of the jaw. J Oral Maxillofacial Surg. 2014;72(10):1938‐1956. 10.1016/j.joms.2014.04.031 25234529

[26] Saad F , Brown JE , Van Poznak C , et al. Incidence, risk factors and outcome of osteonecrosis of the jaw: integrated analysis from three blinded active‐controlled phase III trials in cancer patients with bone metastases. Ann Oncol. 2012;23(5):1341‐1347. 21986094 10.1093/annonc/mdr435

[27] Pimolburtr K , Porter S , Fedele S . Osteonecrosis of the jaw associated with antiangiogenics in antiresorptive naive patient: a comprehensive review of the literature. Biomed Res Int. 2018;2018:1‐14. Article ID8071579. 10.1155/2018/8071579 PMC5937620 29850569

[28] Nicolatou‐Galitis O , Kouri M , Papadopulu E , et al. Osteonecrosis of the jaw related to non‐antiresorptive medication: a systematic review. Supportive Care Cancer. 2019;27:383‐394. 10.1007/s00520-018-4501-x 30353228

[29] Mauceri R , Panzarella V , Morreale I , Campisi G . Medication‐induced osteonecrosis of the jaw in a cancer patient receiving Lenvatinib. Int J Oral Maxillofac Surg. 2019;48(12):1530‐1532. 31378564 10.1016/j.ijom.2019.07.010

[30] Van Cann T , Loysson T , Verbiest A , et al. Incidence of medication‐related osteonecrosis of the jaw in patients treated with both bone resorption inhibitors and vascular endothelial growth factor receptor tyrosine kinase inhibitors. Support Care Cancer. 2017;26:869‐878. doi:10.1007/s00520-017-3903-5 28963584

[31] Wassermann J , Mathy E , Lescaille G , et al. Safety of denosumab in patients with refractory differentiated thyroid cancer and advanced medullary thyroid cancer. J Clin Oncol. 2019;37(15_suppl.e17578):e17578.

